# De Novo Chronic Cluster Headache Following Occupational Inhalational Exposure to Nitric Acid and Nitrogen Oxides: A Case Report

**DOI:** 10.3390/jcm15187255

**Published:** 2026-09-18

**Authors:** Carl H. Göbel, Svea Freitag, Axel Heinze, Katja Heinze-Kuhn, Anna Cirkel, Hartmut Göbel

**Affiliations:** 1 Kiel Migraine and Headache Center, Heikendorfer Weg 9-27, 24149 Kiel, Germany; freitag@schmerzklinik.de (S.F.); heinze@schmerzklinik.de (A.H.); khk@schmerzklinik.de (K.H.-K.); anna.cirkel@uni-luebeck.de (A.C.); hg@schmerzklinik.de (H.G.); 2 Department of Neurology, University Hospital Schleswig-Holstein, Campus Kiel, 24149 Kiel, Germany; 3 Department of Neurology, University Hospital Schleswig-Holstein, Campus Lübeck, 23562 Lübeck, Germany

**Keywords:** cluster headache, case report, nitric acid, nitrogen oxides, occupational exposure

## Abstract

**Background/Objectives:** Nitric oxide (NO) donors reliably provoke cluster headache attacks in susceptible individuals, supporting an important role of NO signalling in cluster headache pathophysiology. Headache following accidental nitric acid inhalation has previously been reported; however, de novo chronic cluster headache following inhalational exposure to nitric acid and nitrogen oxides has not been described. We report a case in which the close temporal relationship and clinical circumstances raise the possibility of secondary headache attribution according to the principles of the International Classification of Headache Disorders, 3rd edition (ICHD-3). **Methods:** We describe the clinical course of a 54-year-old man without a previous history of cluster headache or comparable recurrent headache attacks who developed a novel headache immediately following occupational inhalational exposure involving nitric acid and nitrogen oxides. Clinical characteristics, temporal evolution, diagnostic findings and treatment response were evaluated according to ICHD-3 criteria and interpreted in the context of published evidence concerning nitric acid-induced headache and NO-provoked cluster headache. **Results:** Following the inhalational event in September 2024, a novel headache occurred immediately and subsequently evolved into a characteristic trigeminal autonomic cephalalgia. From December 2024 onward, the patient experienced 5–8 daily attacks of severe to very severe, strictly left-sided periorbital–temporal pain lasting 15–20 min, accompanied by ipsilateral lacrimation and marked restlessness. The disorder persisted without sustained remission for more than one year, fulfilling the phenotypic criteria of chronic cluster headache. Cranial magnetic resonance imaging revealed no structural cause. According to ICHD-3 attribution principles, the de novo onset in close temporal relation to an exposure capable of causing headache supports classification as secondary headache. This attribution is further supported biologically by the established ability of NO donors to provoke cluster headache attacks and activate trigeminovascular mechanisms. **Conclusions:** This case describes a de novo chronic cluster headache phenotype temporally associated with occupational inhalational exposure to nitric acid and nitrogen oxides. The temporal relationship and experimental evidence concerning TRPA1-mediated chemosensory activation, NO–cGMP signalling, CGRP release and trigeminovascular activation provide biological plausibility for a possible exposure-related association. However, neither a toxicological causal relationship nor the proposed transition to persistent trigeminal–autonomic sensitization can be established from this single case.

## 1. Introduction

Cluster headache is a trigeminal autonomic cephalalgia characterized by attacks of severe or very severe, strictly unilateral orbital, supraorbital and/or temporal pain lasting 15–180 min and occurring from once every other day to eight times daily. Attacks are accompanied by ipsilateral cranial autonomic symptoms and/or marked restlessness or agitation. Chronic cluster headache is defined by attacks occurring for at least one year without remission or with remission periods lasting less than three months [1].

Although cluster headache is generally classified as a primary headache disorder, a cluster headache phenotype can occur secondary to another condition. According to the general attribution principles of ICHD-3, when a new headache develops for the first time in close temporal relation to another disorder known to cause headache, or when other criteria for causation are fulfilled, it is classified as a secondary headache attributed to the causative disorder. Importantly, this rule remains applicable when the new headache has all phenotypic characteristics of a primary headache disorder, including cluster headache and other trigeminal autonomic cephalalgias [1].

Nitric oxide (NO) is of particular interest in this context. NO donors have long been known to provoke typical cluster headache attacks in susceptible individuals. Nitroglycerin-induced attacks provide an established experimental model of cluster headache and are associated with activation of the trigeminovascular system [2,3,4,5,6].

A direct clinical association between nitric acid inhalation and headache has also been reported. Granata et al. described headache following accidental nitric acid inhalation. The authors noted that chemical reactions involving nitric acid may generate NO and nitrogen dioxide (NO_2_), providing a potential mechanistic link between inhalational exposure and headache [5].

We report a patient without a previous history of cluster headache who developed a novel headache immediately following occupational inhalational exposure involving nitric acid and nitrogen oxides. During the following months, the headache evolved into a typical and subsequently chronic cluster headache phenotype. To our knowledge, this represents the first reported case of de novo chronic cluster headache following occupational inhalational exposure to nitric acid and nitrogen oxides.

## 2. Detailed Case Description

A 54-year-old man working as a plant and machine operator sustained an occupational inhalational exposure involving nitric acid and nitrogen oxides in September 2024. According to the patient’s history, the event resulted in acute respiratory symptoms requiring emergency medical evaluation and observation for approximately 24 h (Table 1). Quantitative information regarding exposure concentration, exact duration and intensity, the individual nitrogen oxide species involved, respiratory protective measures, and potential co-exposures could not be reliably reconstructed from the available records.

Prior to the occupational event, the patient had no history of cluster headache or comparable recurrent headache attacks. A novel headache occurred immediately following the inhalational exposure. Detailed retrospective characterization of this initial post-exposure headache, including laterality, duration, frequency, and the presence or absence of cranial autonomic symptoms, was not available. Therefore, the precise evolution of individual cluster headache features between September and December 2024 cannot be reconstructed. Persistent consequences of the inhalational event included respiratory and upper-airway complaints, chronic laryngeal discomfort and hoarseness, dyspnoea, olfactory dysfunction and increased sensitivity to irritant odours.

During the months following exposure, the headache phenotype evolved. From December 2024 onward, the patient experienced recurrent attacks of severe to very severe, strictly unilateral left-sided periorbital and temporal pain. Individual attacks lasted approximately 15–20 min and occurred 5–8 times per day. Pain was predominantly stabbing with an additional pulsating quality. Attacks were accompanied by ipsilateral lacrimation and pronounced motor restlessness and occurred particularly during periods of rest, including distressing nocturnal attacks.

No comparable headache or trigeminal autonomic symptoms had occurred before the inhalational event. The attacks continued without sustained remission and subsequently fulfilled the temporal criteria for a chronic cluster headache phenotype.

The inhalational event was also followed by persistent laryngeal and thoracic symptoms. Chronic laryngeal pain was aggravated by speaking and exposure to odours and accompanied by hoarseness. Persistent left-sided thoracic pain was aggravated by physical activity and associated with dyspnoea.

Cranial magnetic resonance imaging performed in October 2025, including intracranial MR angiography, showed no structural intracranial lesion explaining the headache syndrome. Apart from a few nonspecific white-matter signal abnormalities, no relevant pathological findings were identified. Neurological examination revealed no relevant focal neurological abnormality.

Several therapeutic approaches had previously been attempted. Amitriptyline had been used as preventive treatment. Acute medications included sumatriptan, almotriptan, ibuprofen, etoricoxib, paracetamol and metamizole, without adequate long-term control of the headache disorder.

The patient was admitted for specialized multimodal pain treatment from 22 June to 8 July 2026. The clinical attack characteristics and chronic course fulfilled the phenotype of chronic cluster headache according to ICHD-3. Possible attribution to the preceding occupational inhalational exposure was considered separately on the basis of the temporal relationship and the available clinical and experimental evidence.

A five-day course of oral prednisolone (100, 80, 60, 40 and 20 mg/day) produced no significant change in attack frequency. Verapamil retard was gradually titrated under serial electrocardiographic monitoring to a total daily dose of 480 mg. A modest reduction in attack frequency was observed during short-term follow-up.

The patient was instructed in acute treatment using high-flow oxygen and subcutaneous sumatriptan. Lower doses of subcutaneous sumatriptan were insufficient, whereas 6 mg provided satisfactory acute efficacy. Intranasal zolmitriptan was less effective.

The disorder resulted in severe functional impairment. At assessment, the patient had a MIDAS score of 240 and reported marked limitations of occupational, household, social and recreational activities.

## 3. Discussion

The present case describes the de novo occurrence of a headache immediately following occupational inhalational exposure involving nitric acid and nitrogen oxides, followed within approximately three months by the development of a characteristic cluster headache phenotype and subsequently an unremitting chronic course.

The case raises two related questions: first, whether the temporal relationship supports classification as a secondary headache according to ICHD-3; and second, whether a biologically plausible mechanism exists by which nitric acid/nitrogen oxide inhalation might contribute to the development of a persistent trigeminal–autonomic headache disorder.

### 3.1. ICHD-3 Attribution and Secondary Cluster Headache

The distinction between primary and secondary headache is central to this case. ICHD-3 states that when a new headache develops for the first time in close temporal relation to another disorder scientifically documented to be capable of causing headache, or otherwise fulfils criteria for causation, the headache is classified as secondary. Crucially, this remains the case even when the new headache has the characteristics of migraine, tension-type headache, cluster headache or another trigeminal autonomic cephalalgia [1].

In the present patient, there was no pre-existing cluster headache disorder to aggravate. Rather, a novel headache occurred immediately after the occupational inhalational exposure and subsequently evolved into the characteristic cluster phenotype. The sequence was no previous cluster headache → occupational HNO_3_/NO_x_ inhalation → immediate novel headache → persistent/evolving headache → typical cluster headache phenotype from December 2024 → persistent chronic course.

ICHD-3 attribution also requires evidence that the presumed causative disorder or exposure is capable of causing headache; temporal association alone does not generally establish secondary headache attribution. In the present case, such evidence exists [1,5]. Importantly, ICHD-3 attribution based on clinical circumstances should be distinguished from proof of a toxicological causal relationship. In the present case, the close temporal relationship, de novo onset, and published evidence that nitric acid inhalation is capable of causing headache support consideration of secondary attribution, but do not establish toxicological causality.

### 3.2. Evidence That Nitric Acid Inhalation Can Cause Headache

The report by Granata et al. [5] is particularly relevant because it provides direct human evidence linking nitric acid inhalation with headache. The authors reported headache associated with accidental nitric acid inhalation and noted that reactions involving nitric acid may result in the formation of NO and NO_2_. The phenotype in that report was not cluster headache. The case nevertheless demonstrates that nitric acid inhalation is capable of inducing headache in humans.

This distinction is important: the exposure need not previously have been shown to cause exactly the same primary-headache phenotype. Under ICHD-3 attribution principles, a secondary headache may phenomenologically resemble a primary headache disorder [1].

### 3.3. Nitric Oxide as a Specific Experimental Trigger of Cluster Headache

The association between NO signalling and cluster headache provides an additional and more specific level of evidence. Nitroglycerin and other NO donors have been used for decades to provoke cluster headache attacks experimentally. Historical studies showed that nitroglycerin can provoke typical attacks, particularly during active cluster periods, and modern controlled studies have confirmed the reproducibility of this phenomenon [2,3,4,6]. Importantly, occupational inhalational exposure involving nitric acid and nitrogen oxides should not be equated with pharmacological NO donation. The individual nitrogen oxide species and their concentrations were not objectively determined in this patient. Evidence from nitroglycerin provocation studies is therefore used here to support the biological plausibility of NO-related mechanisms in cluster headache, rather than as evidence that an equivalent NO exposure occurred during the occupational event.

Experimentally induced attacks closely resemble spontaneous cluster headache and include characteristic cranial autonomic symptoms. Thus, NO donation is not merely associated with nonspecific headache but can specifically activate biological mechanisms underlying cluster headache. Experimental work also supports activation of trigeminovascular mechanisms and CGRP release during nitroglycerin-induced cluster attacks [2,4,6].

### 3.4. Proposed Pathophysiological Mechanism

**Figure 1:**
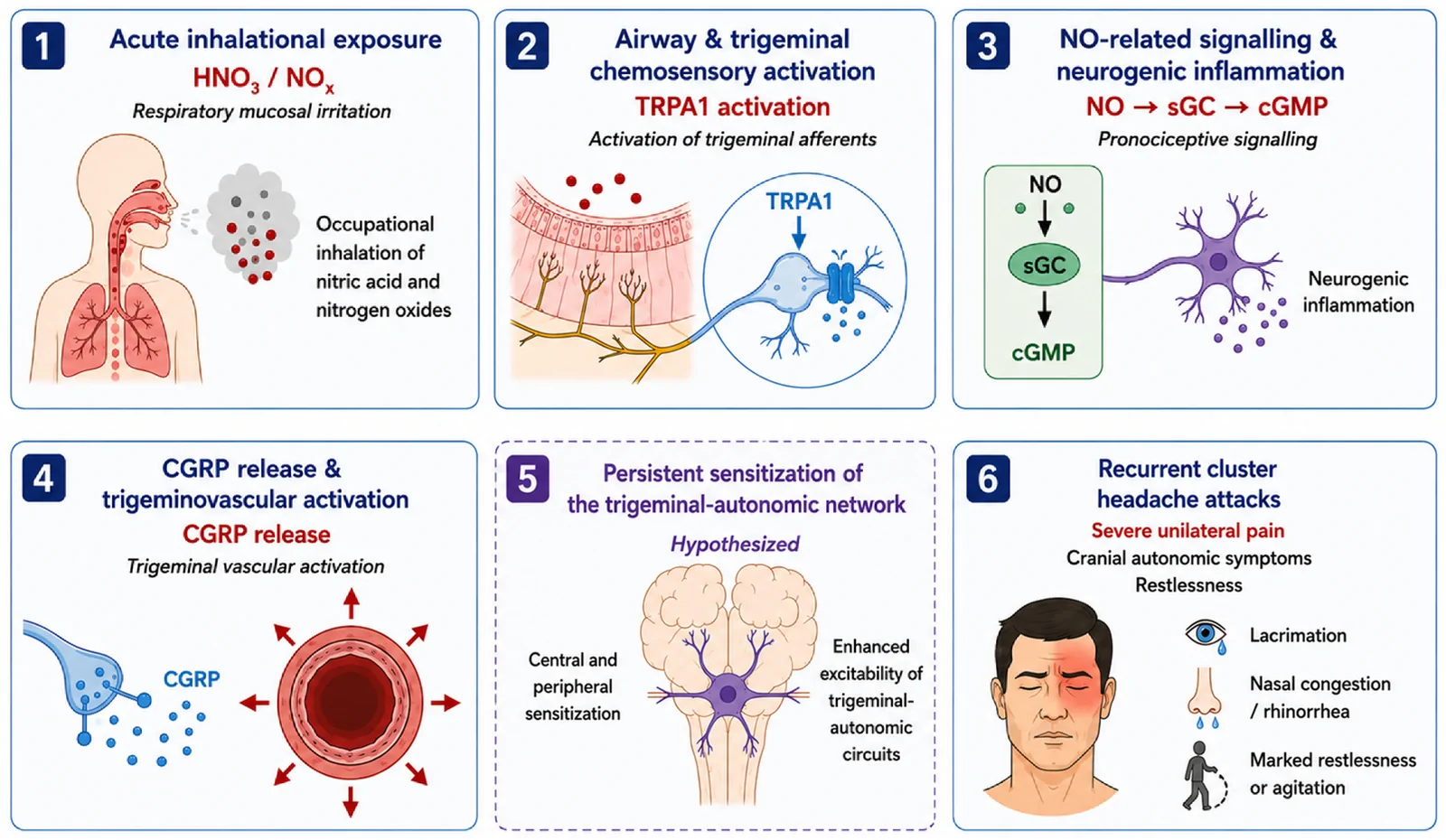
Proposed pathophysiological pathway: from inhalational exposure to chronic cluster headache. Schematic representation of the proposed biological pathway linking acute inhalation of nitric acid and nitrogen oxides to recurrent cluster headache attacks. Inhalational exposure may induce respiratory mucosal irritation and activation of TRPA1-expressing trigeminal chemosensory afferents. Experimental evidence indicates that TRPA1 activation by environmental irritants can induce CGRP release and trigeminovascular activation. In parallel, NO-related signalling through the NO–cGMP pathway may promote neurogenic inflammation and further trigeminovascular activation. Persistent sensitization of trigeminal and trigeminal–autonomic networks is proposed as a potential mechanism for chronification but remains hypothetical. Abbreviations: HNO_3_, nitric acid; NO, nitric oxide; NO_x_, nitrogen oxides; TRPA1, transient receptor potential ankyrin 1; sGC, soluble guanylate cyclase; cGMP, cyclic guanosine monophosphate; CGRP, calcitonin gene-related peptide.

The following mechanistic framework integrates established experimental evidence with biologically plausible inference; none of these mechanisms was directly assessed in the present patient. Based on the clinical observation and experimental literature, we propose that acute nitric acid/nitrogen oxide inhalation may activate two potentially interacting pathways: TRPA1-mediated airway and trigeminal chemosensory activation and NO-related signalling through the sGC–cGMP pathway. Both mechanisms may converge on neurogenic inflammation, CGRP release and trigeminovascular activation. Persistent sensitization of the trigeminal–autonomic network is proposed as a subsequent, hypothetical step leading to recurrent cluster headache attacks (Figure 1).

Nitric acid and nitrogen oxides are potent respiratory irritants. Inhalational exposure may activate chemosensory afferents innervating the respiratory and upper-airway mucosa. TRPA1 (transient receptor potential ankyrin 1), which is expressed on nociceptive sensory neurons, acts as an important chemosensor for a variety of inhaled environmental irritants and provides a potential interface between airway exposure and trigeminal activation. Experimental studies have demonstrated that environmental irritants and TRPA1 agonists stimulate CGRP release from trigeminal ganglion neurons and increase meningeal blood flow following intranasal administration; these responses can be attenuated by TRPA1 or CGRP receptor antagonism [7]. Moreover, intraganglionic signalling between nasal and meningeal afferents has been proposed as a pathway by which inhaled environmental irritants may induce TRPA1-dependent trigeminovascular activation [8]. These findings provide a biologically plausible pathway linking inhalational chemical irritation to activation of the trigeminovascular system [7,8].

A particularly intriguing translational observation is provided by *Umbellularia californica*, commonly referred to as the “headache tree”. Inhalation of its volatile constituents has long been associated with headache, including cluster-like headache attacks. Its principal volatile constituent, umbellulone, is a potent TRPA1 agonist capable of activating trigeminal neurons and inducing CGRP-dependent trigeminovascular responses [9]. This observation further supports the concept that inhaled chemical stimuli may access headache-generating mechanisms through TRPA1-expressing trigeminal afferents.

The persistent laryngeal symptoms, olfactory dysfunction, irritant sensitivity and respiratory complaints observed after the accident support clinically significant involvement of the respiratory and upper-airway system, although they do not themselves establish the mechanism of headache generation.

In addition to this TRPA1-dependent chemosensory pathway, NO can influence nociceptive processing through activation of soluble guanylate cyclase and increased cyclic guanosine monophosphate (cGMP) signalling. In cluster headache, experimental NO donation can provoke typical attacks after a latency, suggesting that its effect extends beyond immediate vasodilatation and involves downstream neuronal and trigeminovascular mechanisms [2,4,6,10].

Activation of trigeminal afferents results in the release of neuropeptides, particularly CGRP, and subsequent trigeminovascular activation. Importantly, Fanciullacci et al. [4] demonstrated increased CGRP-like immunoreactivity in ipsilateral external jugular venous blood during nitroglycerin-provoked cluster headache attacks. CGRP levels returned toward baseline following spontaneous remission or successful treatment with sumatriptan. These findings provide direct human evidence linking NO-donor exposure, cluster headache attacks, and trigeminovascular CGRP release.

The most speculative and potentially most important step is the transition from acute chemical/trigeminovascular activation to persistent susceptibility to cluster headache attacks. Existing NO-donor studies demonstrate that NO can trigger cluster attacks in susceptible individuals; they do not demonstrate that a single intense NO-related exposure can initiate a new chronic cluster headache disorder in a previously unaffected individual. We hypothesize that sufficiently intense inhalational exposure might induce prolonged sensitization or altered excitability within trigeminal and trigeminal–autonomic networks. This proposed transition is hypothesis-generating rather than experimentally established.

### 3.5. Integrating ICHD-3 Attribution and Biological Plausibility

**Figure 2:**
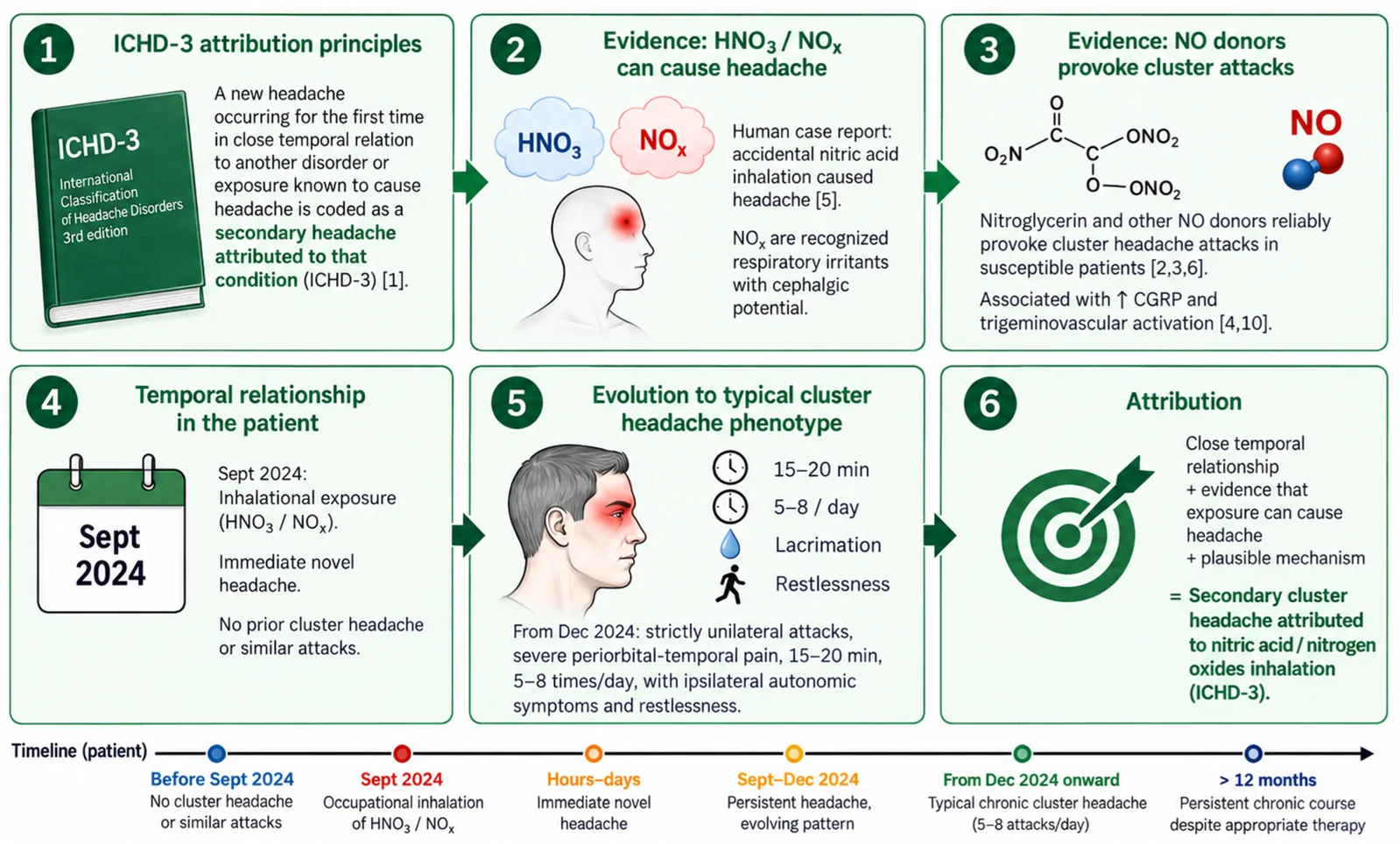
ICHD-3-based attribution pathway: clinical reasoning supporting possible secondary attribution. Schematic representation of the clinical and nosological reasoning supporting attribution of the patient’s de novo cluster headache to occupational inhalational exposure. Headache following nitric acid inhalation has been documented in humans, while NO donors can provoke typical cluster headache attacks; in the present patient, no comparable attacks occurred before exposure, a novel headache developed immediately thereafter, and the disorder subsequently evolved into a typical chronic cluster headache phenotype. Quantitative data concerning the concentration, duration and intensity of exposure to nitric acid and individual nitrogen oxides were not available and represent a limitation of causal assessment. Abbreviations: HNO_3_, nitric acid; NO, nitric oxide; NO_2_, nitrogen dioxide; NO_x_, nitrogen oxides; CGRP, calcitonin gene-related peptide; ICHD-3, International Classification of Headache Disorders, 3rd edition [1,2,3,4,5,6,10].

The clinical–nosological reasoning can be represented as a second sequential pathway: ICHD-3 attribution principles → evidence that HNO_3_/NO_x_ exposure can cause headache → evidence that NO donors specifically provoke cluster headache → close temporal relationship in the patient → evolution into a typical chronic cluster headache phenotype → attribution as secondary cluster headache (Figure 2).

The case is therefore supported by two complementary lines of evidence. The clinical-nosological line is based on de novo onset, close temporal association, evidence that the exposure can cause headache and evolution into a characteristic cluster phenotype. The pathophysiological line is based on the established capacity of NO donors to provoke cluster attacks and activate trigeminovascular mechanisms. Together, these observations support consideration of an exposure-related secondary headache with a chronic cluster headache phenotype. However, coincidental de novo onset of primary chronic cluster headache cannot be excluded.

### 3.6. Differential Diagnosis

The attack phenotype strongly supports cluster headache. The attacks were strictly unilateral and side-locked, severe to very severe, located in the periorbital–temporal region, lasted 15–20 min, occurred 5–8 times daily and were accompanied by ipsilateral lacrimation and marked restlessness. The attack duration lies at the lower end of the ICHD-3 range for cluster headache but remains within the defined 15–180-min interval [1].

Other trigeminal autonomic cephalalgias are less consistent with the clinical phenotype. Hemicrania continua was considered unlikely because the disorder consisted of discrete short-lasting attacks rather than continuous unilateral pain with exacerbations. Other secondary trigeminal autonomic cephalalgias were considered, but cranial MRI, including intracranial MR angiography, did not identify an alternative structural or vascular cause. The attack duration is substantially longer than typical short-lasting unilateral neuralgiform headache attacks, while the frequency and duration are inconsistent with paroxysmal hemicrania. Thus, the principal differential diagnostic issue is not whether the syndrome represents a cluster headache phenotype, but whether it represents spontaneously arising primary chronic cluster headache or secondary cluster headache attributed to the inhalational event.

### 3.7. Limitations

Several limitations must be acknowledged. First, detailed primary documentation of the occupational accident was not available at the time of preparation of this report. The principal limitation for causal attribution is the absence of contemporaneous objective occupational and toxicological exposure documentation. In particular, quantitative measurements of nitric acid, NO, NO_2_ or other nitrogen oxides, exact duration and intensity of exposure, and detailed toxicological measurements could not be reconstructed. The characterization of the inhalational exposure is therefore currently based primarily on the patient’s history and available subsequent medical documentation.

Second, the transition from the initial post-exposure headache to the fully developed cluster headache phenotype occurred over several months. Detailed characteristics of the initial headache and its evolution during this interval could not be reliably reconstructed retrospectively. Although a novel headache developed immediately following the inhalational event, the characteristic pattern of 5–8 daily unilateral periorbital-temporal attacks was established from December 2024 onward.

Third, a single case cannot establish that nitric acid or nitrogen oxide inhalation causes de novo chronic cluster headache in the general population. Coincidental onset of primary cluster headache cannot be completely excluded. Finally, the proposed mechanism of persistent sensitization following acute exposure is hypothetical. Existing experimental studies demonstrate the ability of NO donors to provoke cluster attacks in susceptible individuals, but not the ability of NO exposure to generate a persistent cluster headache disorder de novo.

These limitations notwithstanding, the combination of de novo onset, close temporal association, human evidence that nitric acid inhalation can cause headache, the specific experimental relationship between NO donation and cluster headache, and the subsequent characteristic clinical phenotype provides converging evidence supporting the biological plausibility of an exposure-related association.

## 4. Conclusions

We report a 54-year-old previously free of cluster headache who developed a novel headache immediately following occupational inhalational exposure involving nitric acid and nitrogen oxides. Within approximately three months, the disorder evolved into strictly unilateral periorbital–temporal attacks occurring 5–8 times daily, lasting 15–20 min and accompanied by ipsilateral lacrimation and pronounced restlessness. The syndrome subsequently followed an unremitting chronic course.

According to ICHD-3, a de novo headache developing in close temporal relationship to a condition capable of causing headache is classified as secondary even when its phenotype fulfils the criteria of a primary headache disorder [1]. Previous human evidence demonstrates that nitric acid inhalation can induce headache, while experimental evidence demonstrates that NO donors can specifically provoke cluster headache attacks [2,3,4,5,6,10]. We therefore consider this case to represent a chronic cluster headache phenotype temporally associated with occupational nitric acid/nitrogen oxide inhalation, with a possible secondary attribution according to ICHD-3 principles.

A possible biological pathway involves acute airway irritation and TRPA1-mediated trigeminal chemosensory activation, NO–cGMP signalling, neurogenic inflammation, CGRP release and trigeminovascular activation, followed by persistent sensitization of the trigeminal–autonomic network. However, this single case does not establish a toxicological causal relationship, and coincidental de novo primary cluster headache cannot be excluded. The final transition to persistent sensitization remains hypothetical and requires experimental and clinical confirmation. Recognition of similar cases and systematic assessment of occupational and environmental exposures in patients presenting with de novo trigeminal autonomic cephalalgias may help determine whether this represents an exceptional observation or a previously unrecognized mechanism of secondary cluster headache.

## Figures and Tables

**Table 1:** Clinical Timeline.

Time	Clinical Event	Relevance
Before Sept 2024	No cluster headache or comparable recurrent attacks.	Establishes de novo onset.
Sept 2024	Occupational inhalational exposure involving nitric acid/nitrogen oxides; acute respiratory symptoms; emergency evaluation/observation.	Temporally preceding occupational exposure.
Immediately after exposure	Novel headache began.	Close temporal relationship.
Sept–Dec 2024	Persistent headache with evolving phenotype; detailed characteristics during this interval could not be retrospectively reconstructed.	Transition from acute post-exposure headache to TAC phenotype.
From Dec 2024	Strictly left periorbital–temporal attacks, 15–20 min, 5–8/day, ipsilateral lacrimation and restlessness.	Typical cluster headache phenotype.
Oct 2025	Cranial MRI without structural cause.	Alternative structural secondary cause not identified.
Jun–Jul 2026	Specialized inpatient treatment; prednisolone ineffective; verapamil to 480 mg/day with modest frequency reduction; oxygen and s.c. sumatriptan for attacks.	Therapeutic course consistent with management of cluster headache.
>12 months	Persistent attacks without sustained remission.	Chronic cluster headache course.

## Data Availability

The data presented in this case report are contained within the article. Additional patient-level data are not publicly available due to patient confidentiality and data protection requirements.
